# Endodontic mishaps during root canal treatment performed by undergraduate dental students: An observational study

**DOI:** 10.1097/MD.0000000000027757

**Published:** 2021-11-24

**Authors:** Nuha S. Alghamdi, Youssef A. Algarni, Tasneem Sakinatul Ain, Haifa M. Alfaifi, Alaa A. AlQarni, Jamilah Q. Mashyakhi, Sara E. Alasmari, Muhanned M. Alshahrani

**Affiliations:** a Department of Restorative Dental Sciences, King Khalid University, College of Dentistry, Abha, Saudi Arabia; b Division of Preventive Dentistry, King Khalid University, College of Dentistry, Abha, Saudi Arabia; c King Khalid University, College of Dentistry, Abha, Saudi Arabia.

**Keywords:** dental students, mishaps, obturation, root canal treatment

## Abstract

Endodontic mishaps during root canal treatment (RCT) are considered to be one of the most commonly encountered errors, which affect the quality of treatment and may have dangerous health implications for patients.

The present study was conducted to assess the frequency and types of endodontic mishaps in root canal-treated teeth performed by undergraduate dental students.

A total 404 endodontically treated teeth were performed by undergraduate dental students of King Khalid University College of Dentistry, Abha, Kingdom of Saudi Arabia. The radiographs of the endodontically treated teeth were studied for a period of 6 months, and the related demographic data were collected from patient files.

The most commonly identified mishaps were related to obturation, where the maximum number of cases (68.1%) had under-obturated root canals. More endodontic mishaps were performed by students in level 9 education. The upper left 2nd molar teeth had a higher frequency of mishaps, and molars were found to have more access-related mishaps. Lastly, access-related and instrument-related mishaps had a low frequency of occurrence.

The majority of endodontic mishaps found in the study sample were related to root canal obturation. The undergraduate students at level 9 were less proficient in conducting RCTs with many endodontic mishaps when compared to the cases performed by students at higher levels. The study suggests relevant guidance for dental students while performing RCTs, especially during obturation of the root canals.

## 1 Introduction

Dentists often find it difficult to treat root perforations and other errors occurring during treatment. Several colleges expect their dental students to be proficient in conducting good root canal treatment (RCT) and having a relevant amount of knowledge regarding the prevention of mishaps. Utmost care taken by dental students/dentists while performing RCT is imperative; hence, knowing the common types of endodontic mishaps and the teeth that are more prone to such errors would be beneficial for focused attention and care.

One of the most important roles of endodontic treatment is to remove primary bacterial infection, prevent reinfection, and enable healing of periapical tissues. Endodontic mishaps during root canal treatment are considered to be one of the most commonly encountered errors, which affect the quality of treatment and may have dangerous health implications for patients.^[1]^ RCT refers to the treatment of a tooth that suffers from infected pulp and microbial invasion. Performing RCT ensures that there is no further decontamination of the tooth. Endodontic mishaps are unfortunate mistakes that occur during the treatment or diagnosis of RCT.^[2]^ They can occur due to lack of knowledge, carelessness of the dentist towards giving careful attention, or unforeseen and unpredictable circumstances that are encountered during the surgeries or therapies. All of these relate to endodontic mishaps that can affect prognosis and endanger patients or aggravate the disease even further. These errors in RCT may be related to access opening, in which several types of mishaps occur, such as accidental treatment of the wrong tooth due to incorrect diagnosis or inattention of the dentist, missing the root canal as not all the root canals are easily accessible, damaging the existing tooth with a porcelain crown, accidental perforation, or crown fracture.^[3]^ Some mishaps are concerned with the instrumentation, where at times a ledge is formed in the root canal of the patient's tooth that restricts the penetration of the instrument, and when unnecessarily over-sized files are used, causing apical and/or cervical perforations.^[4]^ During canal preparation, the instrument being used is sometimes separated, mostly because the instrument is stressed or there might be some manufacturing defects, and blockage of the canal may also occur due to the inadequate use of dental clips where the patent canal is disturbed.^[5]^ Other mishaps are under-and over-obturation concerned as well, where the cone is not fitted precisely, nerve paraesthesia or damage with loss of sensation may occur, and vertical root fractures may occur. While these are the most commonly recognised categories of mishaps, other unwanted symptoms or issues such as increased pain, swelling, haemorrhage, tissue emphysema, and accidental dropping of procedural tools or instruments in the oral cavity, all of which are categorised as endodontic mishaps during RCT.^[6]^

To minimise the risk of iatrogenic errors, the American Association of Endodontists published a case complexity assessment form to help general dentists and students manage cases within their competence or refer to advanced cases. Endodontic mishaps may or may not have grave consequences, but there are relevant steps that can be taken to overcome the mishaps with which the patient can regain their oral health. Poor application of RCT procedures usually results in the aforementioned mishaps. Recognition of the mishap and its relevant management procedure, diagnosis, or prognosis is essential to ensure good oral health of a patient.^[7]^ Dentists and dental students may practice prevention techniques to avoid unwanted mishaps. Understanding the causes of such mishaps is essential in developing prevention methods, and helping dentists give more detailed attention to relevant things during RCT treatment. In this regard, the current study was conducted to assess the most common types of endodontic mishaps, the frequency of endodontic mishaps, and teeth prone to such errors among patients treated by dental undergraduate students.

## 2 Methods and analysis

An observational study was planned with a sample of 404 endodontically treated teeth, performed by undergraduate dental students during a period of 6 months from March 2019 to August 2019 at King Khalid University College of Dentistry, Abha, Kingdom of Saudi Arabia. This study evaluated the frequency distribution of endodontic mishaps according to the involved teeth, location of the tooth (maxillary/mandibular), and tooth number, along with the frequency distribution of endodontic mishaps according to students’ educational level 9 (It is decided by the educational council of Saudi Arabia in which, qualifications are grouped together into different levels. Each level corresponds to a particular qualification's degree of difficulty) per year of bachelor of dental surgery. Three periapical radiographs of each root canal-treated tooth were obtained. In addition, the length, taper of root fillings, and the presence of procedural errors were examined. The length of the root canal filling was categorised as adequate, short, or overfilled in relation to the radiographic apex. The procedural errors examined were ledge, missed canal gouging, fractured instruments, and perforation. There were two examiners for the detection of endodontic errors through individual radiographs of endodontically treated teeth. The examiners were calibrated in the Department of Restorative Dental Science, and Kappa statistics were employed to check the inter-examiner reliability.

### 2.1 Sample size

A total of 404 endodontically treated teeth performed by undergraduate dental students is the sample used in this study, through a non-probability sampling technique.

### 2.2 Inclusion criteria

Intraoral periapical radiograph of patients diagnosed with pulp pathology according to the American Association of Endodontics criteria, intraoral periapical radiograph of patients aged between 12 and 40 years, all permanent maxillary and mandibular teeth, teeth prepared with conventional stainless-steel files, and rotary system

### 2.3 Exclusion criteria

Teeth with periapical pathology (such as cysts and tumours), advanced periodontal conditions/perio-endo lesions, root canal treatment performed by postgraduate trainees or faculty members, and previously treated teeth.

The data were organised into Microsoft Excel spreadsheets and statistically analysed using the Statistical Package for the Social Sciences version 19.0 software (Statistical Package for the Social Sciences 19, International Business Machines, Armonk, New York, United States of America. The – chi-square test was used, and *P* < .05, was considered to be statistically significant (*P* < .05).

## 3 Results

**Figure 1:**
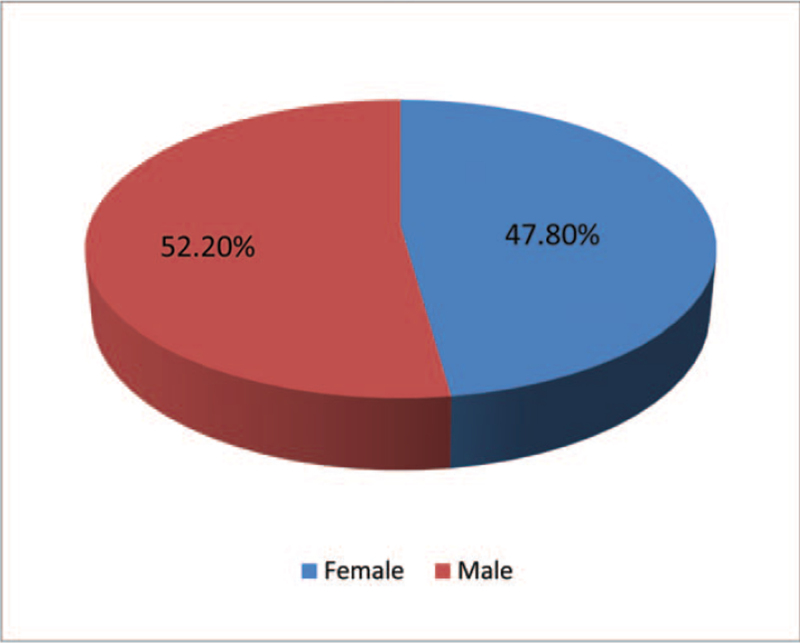
Frequency distribution of teeth according to (a) Patient's Gender.

**Figure 2:**
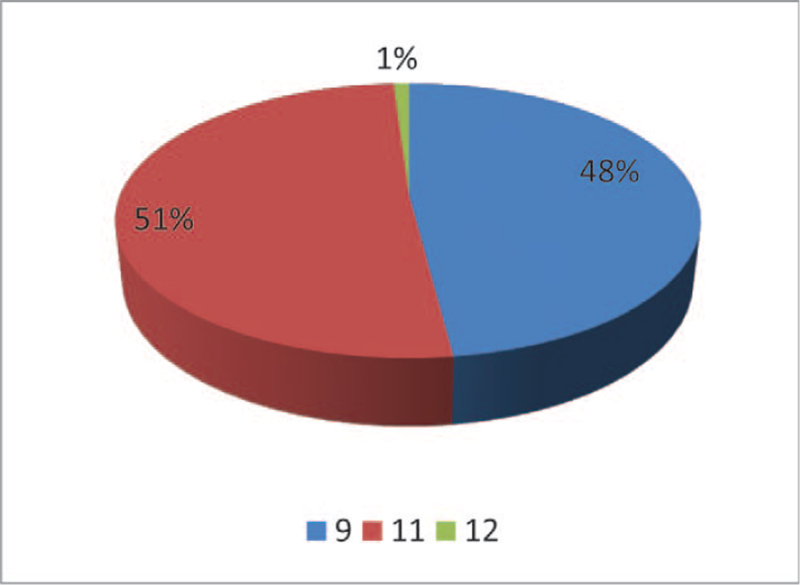
Frequency distribution according to student's level of Bachelor of Dental Surgery course.

Of the total 404 endodontically treated teeth, 211 (52.2%) were male and 193 (47.8%) were female (Fig. 1). 51% of dental students belonged to level 11 (of Bachelor of Dental Surgery course) and 48% of the students were in level 9 (Fig. 2).

Considering the mishaps related to access opening, a majority (86%) of teeth had no mishaps, 11.1% had errors related to gouging, and 2% were related to missed canals. Considering the mishaps related to instrumentation, a majority of 93.8% of the teeth had no errors, 3% faced ledge formation, 1% faced apical perforation, and .5% faced coronal perforation. Regarding the mishaps related to obturation, 68.1% showed under-obturation mishaps, 19.6% had over-obturation mishaps, and the remaining 12.4% had none. It is therefore understood from the analysis that the most commonly identified mishaps were associated with obturation, where the maximum number of cases had under-obturation mishaps. Access-and instrument-related mishaps did not occur frequently. The frequency distribution and comparison of types of mishaps related to obturation with the patient's gender, level of education, teeth, and tooth groups, and in the left and right maxilla and mandible. On applying Pearson chi-square test, male patients were found to have more significant mishaps related to obturation, that is, over obturation (21.3%) and under obturation (71.6%) than females (*P* < .05). The students belonging to Level 9 education showed significantly higher mishaps related to obturation, that is, over obturation (22.2%) and under obturation(59.3%) than other students (*P* < .05). (Table 1).

**Table 1:** Frequency distribution of endodontic mishaps.

Gender	N	%
Female patients	193	47.8
Male patients	211	52.2
Level of education		
Level 9	194	48.0
Level 11	206	51.0
Level 12	4	1.0
Mishaps related to Access opening		
Gouging	45	11.1
Missed canals	8	2.0
None	348	86.1
Perforation	3	0.7
Mishaps related to Instrumentation		
Apical perforation	4	1.0
Coronal perforation	2	0.5
Ledge formation	12	3.0
Mid root perforation	3	0.7
None	379	93.8
Mishaps related to Obturation		
None	50	12.4
Over Obturation	79	19.6
Under Obturation	275	68.1

The frequency distribution and comparison of types of mishaps related to access opening with the gender of patients, level of education of the dental students, teeth, tooth groups, and quadrant (left, right, maxilla, and mandible). On applying Pearson chi-square test, it was found that male patients had significantly higher mishaps related to access opening, that is missed canals (3.8%), perforation (0.9%), and gouging (20.9%) than females, as the *P*-value was less than .05. When compared based on the level of education of dental students, those belonging to level 9 had significantly higher mishaps related to access opening, that is, missed canals (3.6%), perforation (1.0%), and gouging (13.4%). The upper left second molar teeth had significantly higher mishaps related to access opening than the other teeth, that are missed canals (33.3%), perforation (0.0%), and gouging (33.3%). Molar teeth had no significantly higher mishaps related to access opening, that is, missed canals (4.0%), perforation (0.0%), and gouging (8.1%) than other teeth. Maxillary teeth did not show significantly higher mishaps related to access opening, that is, missed canals (1.7%), perforation (0.9%), and gouging (16.4%) than others. (Table 2)

**Table 2:** Frequency, distribution, and comparison of types of mishaps.

		Mishaps related to Access opening and Instrumentation					
Gender		Missed canals	None	Perforation	gouging	Total	*P* value
Female patients	N	0	191	1	1	193	<.001
	%	.0%	99.0%	0.5%	.5%	100.0%	
Male patients	N	8	157	2	44	211	
	%	3.8%	74.4%	0.9%	20.9%	100.0%	
Level of education							
9	N	7	159	2	26	194	.222NS
	%	3.6%	82.0%	1.0%	13.4%	100.0%	
11	N	1	185	1	19	206	
	%	.5%	89.8%	0.5%	9.2%	100.0%	
12	N	0	4	0	0	4	
	%	.0%	100.0%	0.0%	.0%	100.0%	
Teeth							
LL1M	N	1	24	0	1	26	.002∗
	%	3.8%	92.3%	.0%	3.8%	100.0%	
LL1P	N	0	9	0	1	10	
	%	.0%	90.0%	.0%	10.0%	100.0%	
LL2M	N	0	13	0	0	13	
	%	.0%	100.0%	.0%	.0%	100.0%	
LL2P	N	1	13	0	0	14	
	%	7.1%	92.9%	.0%	.0%	100.0%	
LLC	N	0	10	0	3	13	
	%	.0%	76.9%	.0%	23.1%	100.0%	
LLCI	N	0	3	0	1	4	
	%	.0%	75.0%	.0%	25.0%	100.0%	
LLLI	N	0	2	0	1	3	
	%	.0%	66.7%	.0%	33.3%	100.0%	
LR1M	N	0	16	0	2	18	
	%	.0%	88.9%	.0%	11.1%	100.0%	
LR1P	N	0	6	0	2	8	
	%	.0%	75.0%	.0%	25.0%	100.0%	
LR2M	N	0	20	0	0	20	
	%	.0%	100.0%	.0%	.0%	100.0%	
LR2P	N	0	15	0	1	16	
	%	.0%	93.8%	.0%	6.2%	100.0%	
LR3M	N	0	2	0	0	2	
	%	.0%	100.0%	.0%	.0%	100.0%	
LRC	N	0	1	1	2	4	
	%	.0%	25.0%	25.0%	50.0%	100.0%	
LRCI	N	0	5	0	0	5	
	%	.0%	100.0%	.0%	.0%	100.0%	
LRLI	N	0	6	0	0	6	
	%	.0%	100.0%	.0%	.0%	100.0%	
UL1M	N	0	14	0	3	17	
	%	.0%	82.4%	.0%	17.6%	100.0%	
UL1P	N	0	8	1	3	12	
	%	.0%	66.7%	8.3%	25.0%	100.0%	
UL2M	N	0	5	0	1	6	
	%	.0%	83.3%	.0%	16.7%	100.0%	
UL2P	N	1	17	0	6	24	
	%	4.2%	70.8%	.0%	25.0%	100.0%	
ULC	N	0	8	0	0	8	
	%	.0%	100.0%	.0%	.0%	100.0%	
ULCI	N	1	22	0	4	27	
	%	3.7%	81.5%	.0%	14.8%	100.0%	
ULLI	N	0	20	0	2	22	
	%	.0%	90.9%	.0%	9.1%	100.0%	
UR1M	N	3	13	0	2	18	
	%	16.7%	72.2%	.0%	11.1%	100.0%	
UR1P	N	0	23	0	4	27	
	%	.0%	85.2%	.0%	14.8%	100.0%	
UR2M	N	1	1	0	1	3	
	%	33.3%	33.3%	.0%	33.3%	100.0%	
UR2P	N	0	20	1	3	24	
	%	.0%	83.3%	4.2%	12.5%	100.0%	
UR3M	N	0	1	0	0	1	
	%	.0%	100.0%	.0%	.0%	100.0%	
URC	N	0	15	0	0	15	
	%	.0%	100.0%	.0%	.0%	100.0%	
URCI	N	0	21	0	1	22	
	%	.0%	95.5%	.0%	4.5%	100.0%	
URLI	N	0	15	0	1	16	
	%	.0%	93.8%	.0%	6.2%	100.0%	
Tooth group							
Canines	N	0	34	1	5	40	.216NS
	%	.0%	85.0%	2.5%	12.5%	100.0%	
Incisors	N	1	94	0	10	105	
	%	1.0%	89.5%	.0%	9.5%	100.0%	
Molars	N	5	109	0	10	124	
	%	4.0%	87.9%	.0%	8.1%	100.0%	
PreMolars	N	2	111	2	20	135	
	%	1.5%	82.2%	1.5%	14.8%	100.0%	
Teeth types							
Maxillary right	N	4	109	1	12	126	.518NS
	%	3.2%	86.5%	.8%	9.5%	100.0%	
Maxillary left	N	2	94	1	19	116	
	%	1.7%	81.0%	.9%	16.4%	100.0%	
Mandibular left	N	2	74	0	7	83	
	%	2.4%	89.2%	.0%	8.4%	100.0%	
Mandibular right	N	0	71	1	7	79	
	%	.0%	89.9%	1.3%	8.9%	100.0%	

LL1M = lower left first molar, LL1P = Lower left first premolar, LL2M = lower left second molar, LL2P = lower left second premolar, LLC = lower left canine, LLCI = lower left central Incisor, LLLI = lower left lateral incisor, LR1M = lower right first molar, LR1P = lower right first premolar, LR2M = lower right second molar, LR2P = lower right second premolar, LR3M = lower right third molar, LRC = lower right canine, LRCI = lower right central incisor, LRLI = lower right lateral incisor, UL1M = upper left first molar, UL1P = upper left first premolar, UL2M = upper left second molar, UL2P = upper left second premolar, UL3M = upper left third molar, ULC = upper left canine, ULLI = upper left lateral incisor, UR1M = upper right first molar, UR1P = upper right first premolar, UR2M = upper right second molar, UR2P = upper right second premolar, UR3M = upper right third molar, URC = upper right canine, URCI = upper left central incisor, URCI = upper right central incisor, URLI = upper right lateral incisor.

On applying the Pearson Chi-square test, no significant association between mishaps related to instrumentation concerned with the gender of the patient, level of education, teeth, tooth groups, and location of the tooth.

## 4 Discussion

Endodontic procedural errors such as ledges, perforations, blockages, broken instruments, or overfilling could lead to poor prognosis and treatment failure. The assessment of these at the preclinical stages of dental students could highlight aspects that require improvement in endodontic teaching. From the current study analysis, it is observed that the most commonly identified mishaps were related to obturation, wherein the maximum cases had under-obturation-mishaps. Access and instrument-related mishaps were not found to occur frequently, as the majority of the cases (over 80%) did not show any such mishaps. When compared to the literature, a study^[8]^ revealed that several complications are found among patients due to mishaps, where obturation-related mishaps are highly severe and may lead to the maxillary sinus, and over-extension of materials used for filling canals. This finding aligns with the results of the current study. Another interesting study by Al-Anesi et al was carried out to evaluate the obturation quality of extracted teeth by undergraduate Yemeni students. The results of the study revealed that the anterior and single-rooted teeth had significantly better quality than the posterior and multi-rooted teeth which had poor quality root canal fillings. The obturation quality of the mandibular teeth was better than that of the maxillary teeth^[9]^ An assessment study was conducted to assess the technical quality of endodontic cases treated by heterogeneous groups with different clinical experiences. They concluded that clear distinction in clinical experience showed statistical differences in the root filling quality among fourth-year undergraduates, dental interns, endodontic program students, and endodontic specialists.^[10]^ In contrast, in a study among undergraduate dental students, over-instrumentation was found to be the most frequent mishap. The most common error was gouging (access-related) and errors, such as ledge formation due to instrumentation. A study found that access-related errors were highly encountered after root canal treatment, where over-extension of the access cavity was found to prevail in patients. In the current study, there was a statistically non-significant difference in mishaps related to access –opening and instrumentation based on the level of education of students and type/group of teeth. A study found that most patients had one type of mishap, which was also not largely prevalent in the population.^[11]^ These mishaps were concerned with under-filling, gouging, perforation, and poor shaping. While the current research did not find many errors done by the students during RCT, the existing studies^[12]^ have found unacceptable performance and quality of the treatment. Al-Khafaji et al^[13]^ conducted a study to determine the endodontic errors performed by final year undergraduate dental students using radiographs. Errors were less likely to occur during access opening and instrumentation. However, similar to our study, errors such as underfilling and voids linked with obturation were the most frequent.

Practitioners and academicians have acknowledged the concerns associated with endodontic mishaps, and which several technological advancements and the use of instruments made of different materials^[14]^ have been employed to avoid such mishaps. Endodontic errors still occur^[15]^ that correspond to the need for further research to investigate in detail the issues faced by dental students and hence sort out the potential intervention strategies.

The current study did not establish any statistically significant association of the mishaps or differences in results with the type of teeth or teeth groups. However, an observation was made, where the upper left 2nd molar teeth had a higher frequency of mishaps, and molars were found to have more access-related mishaps than the other teeth group. Another study did not find any significant difference in endodontic mishaps among patients with different tooth groups such as canines or anterior teeth.^[16]^ A study found that the lower molars had the highest occurrence of errors and the upper incisors had the lowest level of encountered errors.^[17]^ A study reported the highest frequency of ledge formation and apical transformation in molar teeth.^[18]^ In our study, apical transportation was also quite high in the mandibular teeth. In another study, root canal fillings were not affected by the type of tooth, especially when compared based on maxillary and mandibular teeth.^[19]^ A study on the healing rate of teeth during RCT therapy found that the teeth that most commonly underwent endodontic treatment were premolars.^[20]^ A study evaluated the quality of RCT filling as per teeth and found that premolars had higher quality than other teeth.^[20]^ Another study observed an inadequate RCT quality in molars compared to other teeth.^[21]^

Students’ confidence levels vary according to the year of study and the complexity of the endodontic cases, as well as the practical steps of endodontic treatment. When compared based on the students’ level of education, cases performed by those belonging to level 9 had significantly higher mishaps related to access opening, that is, missed canals (3.6%), perforation (1.0%), and gouging (13.4%) than other students. Cases performed by students in Level 9 education also had more mishaps related to obturation, that is, over obturation (22.2%) and under obturation (59.3%). A study found that final-year students had low self-confidence when treating molar teeth and that they had more confidence when performing basic endodontic procedures in single rooted-teeth.^[22]^ The researchers specified the need for further patient exposure to students. Another study also found a lack of confidence in conducting RCT treatment among students.^[23]^ Another study also evaluated the perceptions of undergraduate dental students regarding their levels of competence and confidence when preparing endodontic access cavities. The majority of the participants indicated their ease of performing endodontic access cavity preparation in anterior teeth, and the lowest confidence levels were indicated on multi-rooted posterior teeth.^[24]^ The technical quality of RCTs performed by preclinical dental students showed that only a few procedures had acceptable technical quality.^[25]^ While the present study did not evaluate the variable of self-confidence, the results of our research can be related to the existing literature,^[26–29]^ where the level of education plays a role in increasing or decreasing the frequency of endodontic mishaps. Several studies have shown that undergraduate students perform endodontic treatments with low technical quality because of insufficient experience.^[30–32]^ However, the occurrence of procedural errors cannot be avoided, even with close clinical supervision. Therefore, it is recommended that more supervised patient exposure be provided to the students to increase their level of knowledge and technical quality of RCT outcomes.

## 5 Conclusion

The majority of endodontic mishaps found in the present study were related to root canal obturation; under-obturation mishaps were the most prevalent. The undergraduate dental students at level 9 were less proficient in conducting RCTs with many endodontic mishaps when compared to the cases performed by students at higher levels. The upper left second molar teeth had a higher frequency of mishaps, and molars were found to have more access-related mishaps. On the basis of the results, the study suggests that universities should incorporate more practical training courses for dental students, where they should be made aware of the most common types of mishaps and ways to avoid or treat endodontic errors upon occurrence. The basic knowledge of dental students related to crown/root morphology of teeth should be given the utmost importance during their formative years of learning, which would result in reducing the risks of endodontic mishaps in the future. Careful and relevant guidance is provided by supervisors to dental students performing RCT, especially during the obturation of the root canals, as it is essential for the successful outcome of the treatment. Moreover, future studies related to endodontic mishaps should be conducted wherein cone beam computed tomography instead of periapical radiographs would be used to diagnose the endodontic errors accurately, and other factors affecting the self-confidence of the students may be included as well.

## Author contributions

**Conceptualization:** Nuha S. Alghamdi.

**Data curation:** Nuha S. Alghamdi, Tasneem Sakinatul Ain, Alaa A. AlQarni.

**Formal analysis:** Nuha S. Alghamdi, Youssef A Algarni.

**Investigation:** Haifa M. Alfaifi, Alaa A. AlQarni, Sara E. Alasmari.

**Methodology:** Sara E. Alasmari.

**Project administration:** Nuha S. Alghamdi, Youssef A Algarni, Haifa M. Alfaifi, Sara E. Alasmari.

**Resources:** Tasneem Sakinatul Ain, Haifa M. Alfaifi, Jamilah Q. Mashyakhi.

**Software:** Youssef A Algarni, Tasneem Sakinatul Ain, Jamilah Q. Mashyakhi.

**Supervision:** Nuha S. Alghamdi, Youssef A Algarni, Tasneem Sakinatul Ain, Jamilah Q. Mashyakhi.

**Validation:** Youssef A Algarni, Haifa M. Alfaifi, Alaa A. AlQarni, Jamilah Q. Mashyakhi, Muhanned M Alshahrani.

**Visualization:** Haifa M. Alfaifi, Muhanned M Alshahrani.

**Writing – original draft:** Nuha S. Alghamdi, Youssef A Algarni, Muhanned M Alshahrani.

**Writing – review & editing:** Nuha S. Alghamdi, Tasneem Sakinatul Ain.
